# Novel semi-automated algorithm for high-throughput quantification of adipocyte size in breast adipose tissue, with applications for breast cancer microenvironment

**DOI:** 10.1080/21623945.2020.1787582

**Published:** 2020-07-07

**Authors:** Frank L. Lombardi, Naser Jafari, Kimberly A. Bertrand, Lauren J. Oshry, Michael R. Cassidy, Naomi Y. Ko, Gerald V. Denis

**Affiliations:** a Department of Biomedical Engineering, Boston University, Boston, MA, USA; b BU-BMC Cancer Center, Boston University School of Medicine, Boston, MA, USA; c Slone Epidemiology Center, Boston University School of Medicine, Boston, MA, USA; d Section of Hematology-Oncology, Boston Medical Center, Boston, MA, USA; e Department of Surgery, Boston Medical Center, Boston, MA, USA; f Department of Pharmacology and Experimental Therapeutics, Boston University School of Medicine, Boston, MA, USA

**Keywords:** metabolism, cancer risk, algorithm, MATLAB, image analysis

## Abstract

The size distribution of adipocytes in fat tissue provides important information about metabolic status and overall health of patients. Histological measurements of biopsied adipose tissue can reveal cardiovascular and/or cancer risks, to complement typical prognosis parameters such as body mass index, hypertension or diabetes. Yet, current methods for adipocyte quantification are problematic and insufficient. Methods such as hand-tracing are tedious and time-consuming, ellipse approximation lacks precision, and fully automated methods have not proven reliable. A semi-automated method fills the gap in goal-directed computational algorithms, specifically for high-throughput adipocyte quantification. Here, we design and develop a tool, AdipoCyze, which incorporates a novel semi-automated tracing algorithm, along with benchmark methods, and use breast histological images from the Komen for the Cure Foundation to assess utility. Speed and precision of the new approach are superior to conventional methods and accuracy is comparable, suggesting a viable option to quantify adipocytes, while increasing user flexibility. This platform is the first to provide multiple methods of quantification in a single tool. Widespread laboratory and clinical use of this program may enhance productivity and performance, and yield insight into patient metabolism, which may help evaluate risks for breast cancer progression in patients with comorbidities of obesity.

**ABBREVIATIONS:** BMI: body mass index.

Breast cancer incidence, progression and mortality continue to pose a serious public health challenge, particularly among underserved patients and in safety net hospitals [1–3], where the prevalence of comorbid obesity and metabolic disease are also high [4]. Recent appraisals of expected numbers of new cases of breast cancer in the United States report estimate 271,270 diagnoses annually, of which about 205,670 will be invasive breast carcinoma in women [5]. Furthermore, breast cancer accounts for 30% of all new cancer diagnoses in women and was projected in 2019 to account for 41,760 of U.S. cancer deaths in women [5]. Black women continue to have higher breast cancer mortality rates than white women [6] and both race and lower socioeconomic status continue to be strongly associated with U.S. cancer mortality [5,7-10].

It has been long appreciated that obesity and metabolic disease disproportionately affect underserved patients [11,12], for whom access to high-quality nutrition and regular physical activity may be limited [13]. In obesity, low-grade chronic inflammation, adipocyte dysfunction and metabolic abnormalities alter the breast tumour microenvironment and upregulate expression of aromatase [14] to exacerbate risk for breast cancer incidence and progression. We have investigated chronic metabolic disease, particularly Type 2 diabetes in the context of obesity [15], which associates with increased adipocyte size [16] and increased incidence, progression and mortality in breast cancer [17,18]. Although obesity is a well-established risk factor for incidence of oestrogen receptor-positive breast cancer in post-menopausal women, there is also a significant positive association between obesity-driven Type 2 diabetes and incidence and progression of oestrogen receptor-negative breast cancer [17]. We are currently participating in a large national trial (NCT02750826) to investigate the effect of a supervised weight loss intervention on breast cancer outcomes, in overweight and obese women with stages II and III HER-2 negative breast cancer. Thus, the relationships among BMI, metabolism, inflammation and incidence or outcome for different subtypes of breast cancer are not fully understood; these investigations continue to have high significance.

Although transcriptional profiling of patient primary tumour tissue [19], or the 21-gene recurrence score (Oncotype Dx) for oestrogen receptor-positive, early-stage breast cancer [20], have proven utility for clinical decision-making, these tools may not always be available, affordable or used in a timely fashion in the safety net hospital setting [21,22]. Furthermore, the seminal study (Trial Assigning Individualized Options for Treatment or TAILORx) prospectively validating the Oncotype Dx Recurrence score in hormone receptor-positive, HER-2-negative, node-negative breast cancer, included only 7% Black patients. A recent analysis of race and clinical outcomes in this trial demonstrated inferior outcomes for Black patients despite similar recurrence scores and comparable systemic therapy [23]. This result supports the contention that factors beyond the genomic landscape are critical in determining worse outcomes in Black women.

We have used antibody capture assays to profile plasma cytokines of metabolically at-risk patients with obesity to develop signatures of cytokines known to drive breast cancer progression and metastasis [24]. Yet, we have found that vulnerable patients are often reluctant to undergo blood draws required for these novel tools [25]. Furthermore, blood profiling of metabolic and inflammatory analyses is not covered by insurance, nor are they included in the standard of care for breast cancer patients with metabolic co-morbidities. Thus, there is an unmet need for inexpensive, robust pathology measures to supplement routine clinical information, to assist in potential clinical decision-making and risk assessment for breast cancer patients. New pathology tools to characterize adipocyte dysfunction in obesity may help identify and stratify the highest risk patients, and assist clinical decision-making to reduce mortality.

Analysis of adipocyte size distribution in adipose tissue samples has been a popular practice for researchers looking to use histological samples to gain insight into metabolic and immunological conditions [26–29]. Adipocyte size, shape and contour are highly irregular in most human histological samples, thus experience and skill are required to distinguish bona fide adipocytes from artefacts introduced in the fixation and staining process or tissue subtypes not of interest. Furthermore, adipocyte measurement must be comprehensive and unbiased, else the calculated size distributions will not be representative of the sample and metabolic diagnosis will be inaccurate. Additionally, in the clinical pathology laboratory, the measurement methods must be well suited for high volume cell quantification and reproducibility. Thus, repeatability and precision must be a primary focus for assessing the utility of better methods. Lastly, if adipocyte quantification remains inefficient, bandwidth amongst clinical pathologists, thus longer turnaround times, may hinder broader access for all patients whose care may otherwise benefit from such information.

Comparison of current adipocyte quantification methodology from digital histological images reveals two primary, well-established methods and one emerging method: hand-tracing and ellipse approximation have been widely used and described for adipocyte quantification, and fully automated image analysis remains an emerging technique [27,30,31]. The hand-tracing method, manual by definition, is considered the gold standard for clinical and research practice because of its accuracy and reproducibility, and thus remains the current convention for obesity and metabolism research studies. An experienced user traces the membrane of the adipocyte, avoiding artefacts or irrelevant tissue, as well as extrapolating missing or deteriorating membrane boundaries. The cell area is calculated by scaling the pixel area to relevant units (μm^2^). The time required to perform these analyses using the hand-tracing method is largely proportional to the number of cells quantified. For large volume or high-throughput sampling, the method is highly inefficient and time-consuming. Overall, the utility of the hand-tracing method for high-throughput adipocyte quantification is limited because it is tedious, inefficient and dependent on the skills of the operator, which introduce human error and subjectivity [31].

The ellipse approximation method is also described in immunology research publications [30], whereby the area of a superimposed ellipse over the adipocyte is calculated to approximate the area of the cell. The user measures the semi-major (A) and semi-minor (B) axes of the adipocyte and then calculates the area using the equation, **Area** = π*A*B [30]. However, depending on the user’s estimation of the appropriate axes, the ellipse approximation may severely underestimate or overestimate the true size of the cell. The accuracy of this method is correlated with the irregularity of adipocyte size, shape and contours and user subjectivity. Although an ellipse approximation is less manually intensive for the user and may be slightly more efficient, it has limitations as described and remains a non-exact methodology.

The third method that has been and continues to be explored in the literature as an emerging technique, fully automated quantification, seeks to enable high-throughput measurement of adipocytes without, or with minimal intervention from a user. Such tools leverage multiple layers of image manipulation, processing such as converting to binary and setting an intensity threshold, and segmentation to calculate adipocyte size by the area contained in the boundary detected and subtracted from the background [30–32]. Although these approaches have the potential to be efficient, they often exhibit technical complications and unreliable performance when used to analyse histological samples of sub-optimal quality. A challenge with fully automated quantification of adipocytes lies in favourably modulating the signal-to-noise ratio of adipocyte cell boundaries with artefact. Key limitations of this method include improving the ability to detect cell membranes and to account for intra-sample heterogeneity of adipocyte quality, signal-to-noise and lighting conditions [32]. Fully automated methods have not proven reliable with diverse image samples [32] and our group was unable to reliably produce outputs using these tools with image samples outside of samples provided by authors. Therefore, and considering the literature, we do not determine fully automated methods of adipocyte quantification to be standard laboratory practice at this time.

Early and current methodology of adipocyte quantification are problematic and insufficient, and fail to reach a compromise between accuracy, efficiency and robustness, which affects the productivity of the user and the potential of adipocyte quantification as a prognostic indicator [28,29]. With these considerations in mind, we chose to design an interactive software tool, developed in MathWorks® MATLAB, which quantifies adipocytes and relays data in a clear and informative manner. The goal of tool was to provide investigators a semi-automated method of adipocyte quantification and enhance productivity over currently standard methodology, as well as offer the ability to use other widely used methods as determined by sample type, source, condition or intra-sample heterogeneity. The tool, AdipoCyze, incorporates (i) a novel, semi-automated tracing algorithm to follow the membrane of adipocytes precisely, along with benchmark methods, such as (ii) manual tracing and (iii) ellipse approximation.

Here, we describe the development of AdipoCyze and use the tool to assess the accuracy and speed of adipocyte quantification using the novel, semi-automated tracing algorithm, compared with methods of standard practice, hand-tracing and ellipse approximation.

A semi-automated method that allows the user to select cells of interest with ‘one click,’ while all measurements, calculations and renderings are performed through computational algorithms, may fill the need in adipocyte quantification and offer a solution to the challenges described. By enabling high-throughput quantification of both optimal and sub-optimal histological samples, minimizing errors, improving accuracy and increasing efficiency, we expect this novel approach to enhance the productivity of the user.

The availability of and widespread adoption of AdipoCyze may usefully inform the standard of care in risk management of obesity-associated cancers, as well as other clinical approaches where adipocyte size measurements are beneficial, such as after bariatric surgery. The tool is well suited to low-resource settings and could assist with prognosis and risk stratification for vulnerable or underserved breast cancer patients, who suffer from increased co-morbidities including obesity, diabetes and hypertension.

## Materials and methods

### Human subjects

The Komen Tissue Bank collected detailed medical history, including diagnoses and medications, from a non-clinical, volunteer population. This information was obtained for each subject in the analysis, stripped of HIPAA identifiers and used with written permission from the Komen Tissue Bank. The donated samples were collected with informed consent following Helsinki guidelines. Protocols, including laboratory procedures used in the study, were submitted to and approved by the Institutional Review Board, Boston University. The data are archived in the Virtual Tissue Bank (https://virtualtissuebank.iu.edu/), which is accessible to the general public. Self-reported race of all subjects recruited to this study was African American; all subjects were female. These samples provided a range of sizes to permit the computational algorithm to be developed and tested across a diverse set of samples with varying degrees of quality in sample preservation.

### Image acquisition and analysis

We obtained breast histological sections that included adequate numbers of adipocytes from a panel of volunteers drawn from the general community, who donated specimens to the Susan G. Komen for the Cure Tissue Bank (KTB) at the Simon Cancer Centre (Indiana University). Sections had been stained with H&E and captured as digital images by microscopy. Full-slide digital images of histological breast adipose samples obtained from Komen for the Cure were imported as.svs files, native to Imagescope software. The image files were converted to.tiff and subsequently.jpeg to maintain a high quality and adequate compression for computational analysis. Samples of regions of interest were segmented from each image at constant magnification.

### AdipoCyze design approach

AdipoCyze was developed using MathWorks MATLAB suite R2013b. Functional requirements included an intuitively designed, interactive, graphic user interface (GUI), a simple process to import and transform histological image files, the ability to analyse the image using three available quantification methods such as, Trace mode (TM), hand trace (HT), and ellipse approximation (EA), and the ability to export results upon the completion of analysis. The program was constructed to export results in relevant file formats for tertiary analyses (.xls.,jpg.,fig.,mat). The final program executable file (.exe) was compiled using MATLAB Runtime Complier for distribution and execution on PCs with or without MATLAB installed.

### Semi-automated trace mode and algorithm design

The semi-automated cell adipocyte quantification method was designed to allow the user to select cells of interest with ‘one click,’ while all measurements, calculations, and renderings were to be performed through computational computer algorithms. We designed a user experience such that in the Tracing Mode, the user clicks the approximate centre of the cell of interest and the algorithm traces the interior perimeter of the cell using a recursive tracing algorithm. The interior cross-sectional area of the cell is then calculated in square pixels, the calculated region is displayed and the cell of interest is labelled for analytical purposes. In order to use the trace mode with one-click tracing algorithm, the image of interest is required to be transformed using the default transformation parameters in AdipoCyze or manually, following the three-step process of modulating low levels and shadows, converting to binary, and despeckling.

### Hand-trace mode design

We sought to design a hand-trace mode that mirrored the conventional method to be included into AdipoCyze. In this mode, the user is able to manually trace the perimeter of the cell of interest by selecting its vertices by hand. The drawn area is dynamically displayed such that the user is able to visualize the area enclosed. The interior cross-sectional area of the cell is then calculated in square pixels and the cell is labelled for analytical purposes.

### Ellipse approximation mode design

The goal of the ellipse approximation module was to provide users with the option to use the ellipse approximation method to quantify adipocytes, should the analysis or goal of research demand. In this regard, we developed an analysis module such that the user measures the major axis of the ellipse by assessing the size, curvature and orientation of the adipocyte. After the drawing of the major axis, a perpendicular line is projected from the centre of the cell to allow the user to define the semi-minor axis. The interior cross-sectional area of the ellipse approximation is then calculated in square pixels using the equation, π*A*B, where A and B are the semi-major and semi-minor axes, respectively. Cells are labelled for analytical purposes.

### Adipocyte quantification

Cells 1–15 of the sample were analysed, in order, three times over using each of the adipocyte quantification methods available from the AdipoCyze tool. All cells were measured in the same order and time in between measurements of each cell was recorded in seconds.

#### Hand trace

Cells were measured manually by clicking on the perimeter of the cell to create the first vertex of the polygonal area. The user double-clicked to complete the measurement of an individual cell. Timing was measured from the point at which the initial image loaded in AdipoCyze and analysis can begin to the point in time when the full analysis of the 15 cells was complete. Splits of analysing individual cells were also measured.

#### Ellipse approximation

Cells were measured by approximating an elliptical area over the cross-sectional area of the adipocyte of interest. The user clicked the vertices of the cell to measure the major axis of the ellipse where then a semi-minor axis was drawn perpendicular and through the centre. The area of the ellipse was calculated with the equation: π*A*B, where A and B are the semi-major and semi-minor axes, respectively. Timing was measured from the point at which the initial image loaded in AdipoCyze and analysis can begin to the point in time when the full analysis of the 15 cells was complete. Splits of analysing individual cells were also measured.

#### Tracing algorithm

Analysis using the trace mode first required the sample image to be transformed; the following parameters were applied: Low Level in: 0.9; Shadows: 0.15; Binary Threshold: 0.8; Despeckle Pixel Radius: 5; Number of Passes: 5. To measure cells using the tracing algorithm, the user clicked anywhere in the interior of a cell of interest. The tracing algorithm finds the interior boundary of the cell membrane and traces the interior perimeter of the cell to measure cell perimeter and cell area. Utilizing the tracing algorithm requires transforming the image in AdipoCyze. Time was measured from the point at which the initial image loaded in AdipoCyze and image processing was initiated to the time when the full analysis of the 15 cells was complete. Splits of analysing individual cells were also measured.

## Results

### Intra-sample heterogeneity

**Figure 1.**
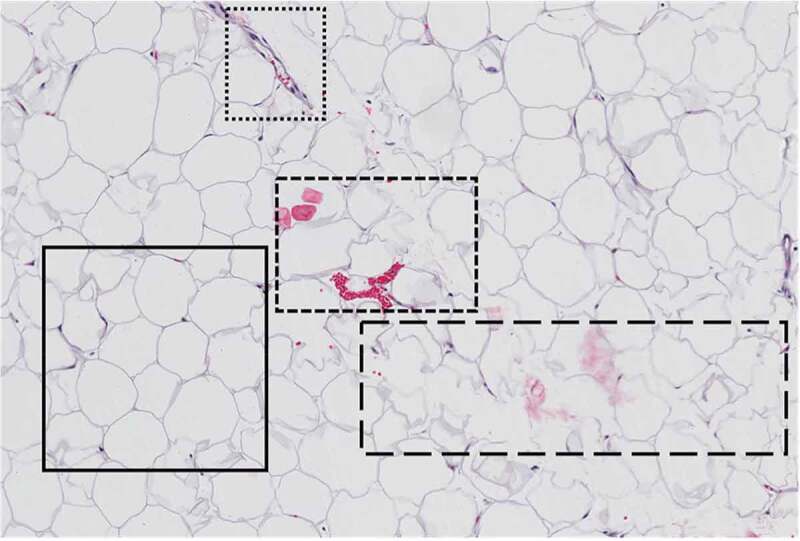
Representation of intra-sample heterogeneity. The area enclosed in the solid-line border represents an optimal sample containing adipocytes characteristically shaped with structured cell membranes, well-defined borders and absence of cellular or foreign artefacts. Sub-optimal samples are represented in the subsequent enclosed sections. The area enclosed in the dotted line border and the area enclosed in the dashed line border depict the presence of morphological signatures and cellular artefacts. The area enclosed in the long-dash border contains poorly defined adipocyte cells with deteriorated cell membranes indicative of poor fixation or non-uniform slices that are too thin for adipose tissue

Samples from the Komen Tissue Bank were analysed and yielded a wide variety of intra-sample heterogeneity, representing a variety of sample quality. As shown in Figure 1, within a single field of view, the quality of adipocytes for measurement can vary. The area enclosed in the **solid-line** border represents an optimal sample containing adipocytes characteristically shaped with structured cell membranes, well-defined borders, and absence of cellular or foreign artefacts. Sub-optimal samples are represented in the subsequent enclosed sections. The area enclosed in the **dotted line** border and the area enclosed in the **dashed line** border depict the presence of morphological signatures and cellular artefacts. The area enclosed in the **long-dash border** contains poorly defined adipocyte cells with deteriorated cell membranes, indicative of poor fixation or nonuniform slices that are too thin for adipose tissue.

### AdipoCyze output

**Figure 2.**
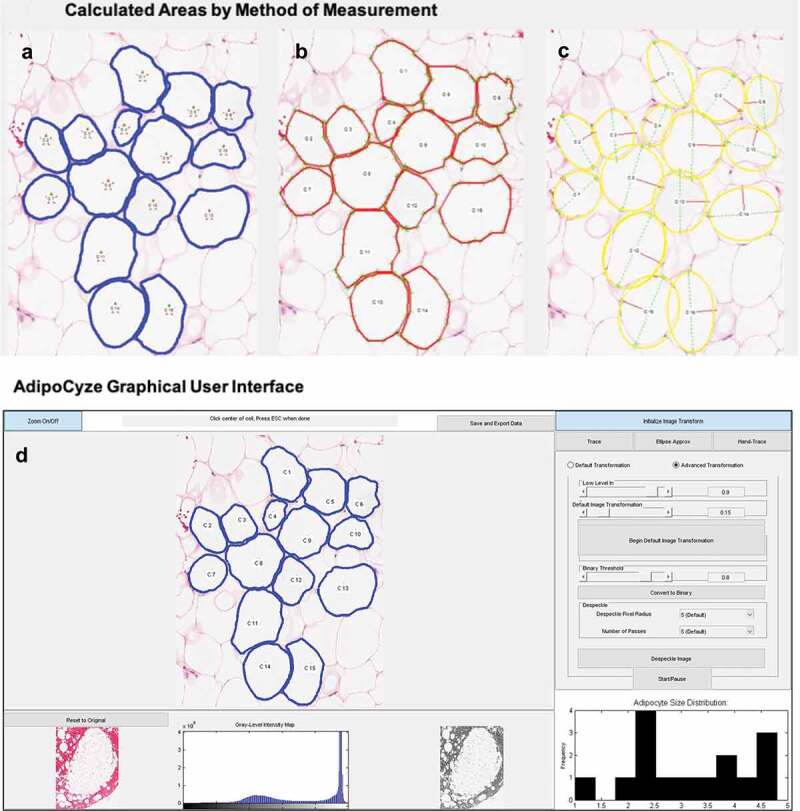
Compares the calculated areas for each measurement method. (a): Measurements with the TM method are outlined in blue. (b): Measurements with HT method are outlined in red. (c): Measurements with the EA method are outlined in yellow with the calculated major axis is shown as a green line and semi-minor axis is shown in the red line. (d): AdipoCyze Graphical User Interface (GUI) shown in TM

The output of analysis performed using AdipoCyze can be seen in Figure 2(a–c) which visually compares the cell boundaries and calculated areas for each measurement method. Cells are numbered in the order analysed. Measurements with the Trace Mode (TM) (Figure 2(a)) algorithm are shown in blue outline and notably most thoroughly trace the contours of the adipocytes and manage to account for irregularity in shape and size. Measurements with Hand Trace (HT) method (Figure 2(b)) are outlined in red, where the green crosses represent the cell vertices selected for each cell. The outlined perimeters are markedly more polygonal, composed of straight lines, in contrast to the other two methods. Measurements with the Ellipse Approximation (EA) method (Figure 2(c)) are outlined in yellow. The major axis is shown as a green line and semi-minor axis is shown in the red line. Apparent in the analysis of the EA method is the correlation in approximation with the regularity of adipocyte shape. Ellipses drawn for cells C1 and C14 appear to underrepresent the true size of corresponding adipocytes, whereas C9 and C12 appear to over-represent the size. Additionally, a number of ellipse approximations demonstrated overlap as shown in (C3, C8), (C4, C9), and (C15, C16).

).

The AdipoCyze Graphical User Interface (GUI) in trace-mode is shown in Figure 2(d), depicting the output of our holistic development efforts. The resulting comprehensive AdipoCyze tool features a prominent interactive analysis window with zoom capability, a grey-level intensity map of the loaded image, and a status-bar with instructions for the user. The right panel allows the user to perform image transformation to increase signal as well as select as the desired method for quantification. A dynamic histogram of adipocyte size distribution is drawn as cells are measured.

### Adipocyte area calculation by method

**Figure 3.**
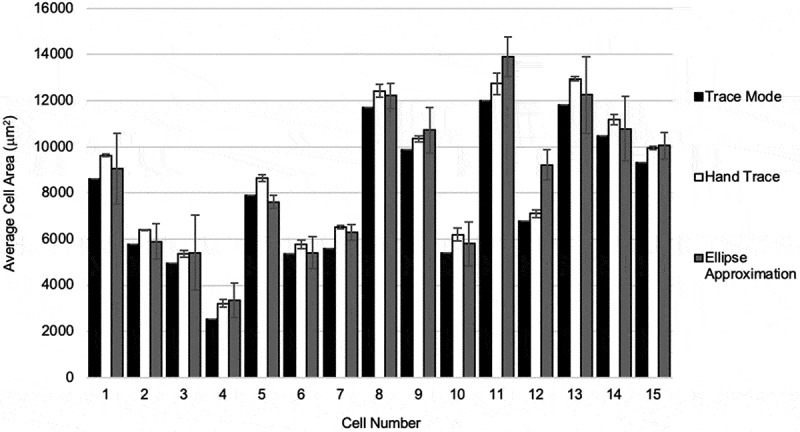
Comparison of adipocyte area calculation by method by cell. Results of analysing adipocyte size (μm^2^) by cell, in the order of cells measured are shown. Each data point in the bar chart represents the average of three repeated runs of for a measurement method. Measurements using the trace mode, hand trace, and ellipse approximation methods are shown in black, white, and grey bars, respectively. Error bars represent standard deviation of measurements for individual cells

Each cell was analysed three times using each analysis method. The results of analysing adipocyte size (μm^2^) by cell, in the order of cells measured are shown in Figure 3. Each data point in the bar chart represents the average of three repeated runs for a measurement method. Cells analysed by the TM were consistently measured to a smaller area than HT and EA. The magnitude of underestimating the area of adipocytes using the TM compared to the HT method was also consistent. As the HT method is the reference, there was variability in over-estimating and under-estimating cell area when comparing HT and EA methods where the EA method over-estimated the size of the adipocyte in cells 9, 10, 12, and 16, and underestimated the size of the adipocyte in the other cells. Overall there was uniformity in area measurements across methods. The variation between repeated measurements was highest with the EA method, followed by the HT method, and no variation with the TM method.

### Adipocyte size distribution by method

**Figure 4.**
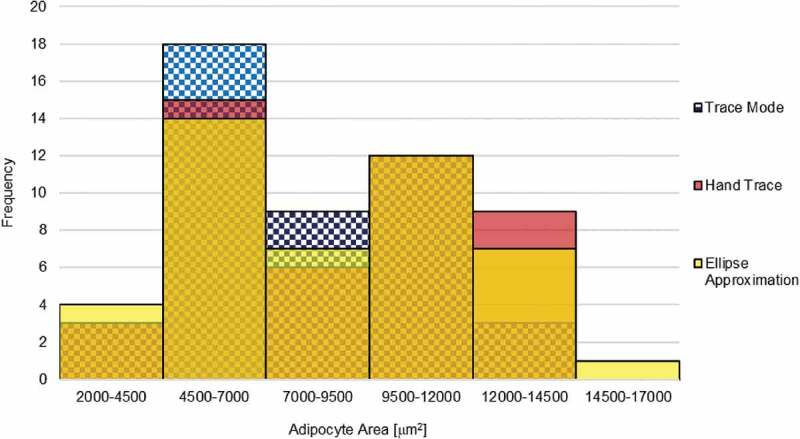
Adipocyte size distribution by method. Results from various methods are shown in 100% overlap to show the shape of distribution across 6 bins. Adipocyte area size distribution from the TM, HT, and EA is shown in blue check pattern, red, and yellow, respectively. Overlap between distributions is shown by secondary shades

The distribution of adipocyte size using the TM, HT, and EA methods is demonstrated in the histogram in Figure 4. The adipocyte size distribution shows a left bias as well as a bimodal distribution. The TM mode yielded higher frequencies of smaller calls between 4500–7000 μm^2^ and 7000–9500 μm^2^ bins, and is visually shifted left slightly compared to HT and EA methods. The largest measurement was taken using the EA method.

### Concordance

**Figure 5.**
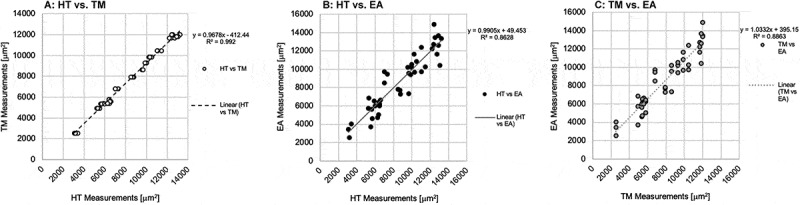
Concordance between adipocyte quantification methods was analysed and be observed in the scatter plots (a, b, c). Variables were plotted along the X, Y axes. Linear trendlines were plotted through each series. Concordance was evaluated based on slope and correlation coefficient of trendline. (a): Concordance of HT with the TM is observed from the series with white circles and black outline. (b): Concordance of HT with EA is observed in the series of black circles. (c): Concordance of the TM and the EA is observed in the series of grey circles

Concordance was evaluated based on slope and correlation coefficient of trendlines in Figure 5. HT and TM demonstrated high concordance both visually and mathematically with a strongly linear scatter, a slope of 0.97 and R^2^ of 0.992. The negative intercept in the linear regression confirms observations from Figure 3 of TM underestimating adipocyte areas relative to HT. Plotting HT and EA suggests a broad scatter but the slope of 0.99 in Figure 5(b). The correlation coefficient of R^2^ = 0.86 reveals a weaker correlation compared to (HT, TM) Figure 5(a) and (TM, EA) Figure 5(c). The positive intercept of the linear regression confirms observations from Figure 3 of EA overestimating adipocyte areas relative to HT. Concordance of the tracing mode and the ellipse approximation shows a scatter between Figure 5(a,b). The slope of 1.03 shows a positive correlation, while the R^2^ of 0.89 reveals a correlation weaker than that of (HT, TM) but stronger than that of **(HT, EA)**. The positive intercept of the linear regression confirms observations from Figure 3 that EA greatly overestimates adipocyte areas relative to TM. Generally, all three methods were generally concordant with each other. While the slope of HT vs TM is <1, the R^2^ value was the highest at 0.992 demonstrating the strongest explanation of the data by the linear fit.

**Figure 6.**
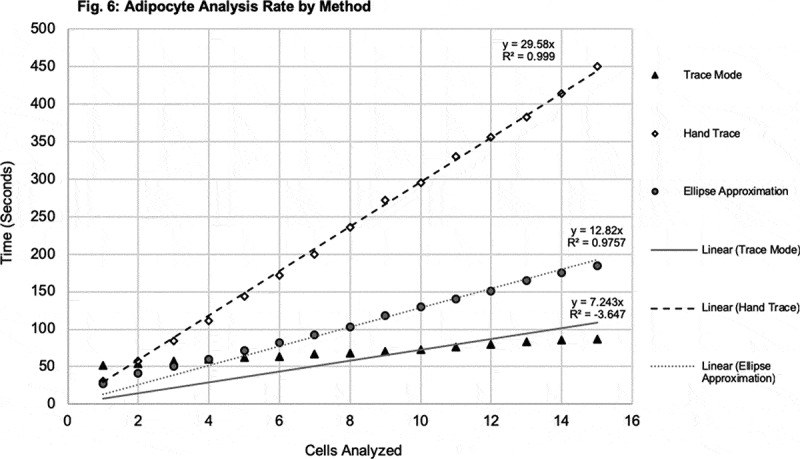
Adipocyte analysis rate by method. The average time to measure each cell was determined amongst the different methods. Average time to measure each cell using TM, HT, and EA, are shown plotted against each cell with symbols, black triangle, white diamond, and grey circle, respectively. Linear trendlines are displayed on each series of data

### Rate of analysis

The HT and EA methods took the longest time to complete, measuring 15 cells. The TM method took less than one-quarter of the time to complete measuring cells in comparison to the HT method, and nearly half than that of the EA. The time to measure the first cell was similar between the HT and the EA methods, and the longest with the TM. Linear trendlines are displayed on each series of data. Both the HT and EA methods demonstrated evidence of a linear relationship, whereas the TM showed evidence of a non-linear relationship with time, likely due to the time spent upfront to transform the image. Extrapolating the results shown here for the measurement of 15 cells, it would be expected that the gap between time to measure using TM and HT and EA methods increases with higher volumes of cells.

### Measurement of ideal and non-ideal adipocyte images

When specifically comparing the measurement methods in a heterogenous adipocyte image sample in Figure S1, we saw that both the ideal cell and the non-ideal cell were able to be measured with each method. Visual inspection of the regions calculated revealed that TM was able to closely trace the interior contour of the ideal cell and closely resembles the region calculated from hand-tracing (Figure S1d). Consistent with other observations, the ellipse approximation method appears to over-estimate the cell area as the region calculated from ellipse approximation by including regions in the area calculation that are not part of the cell (Figure S1c). When non-ideal cells are measured, we see higher variability in measurement across methods (Figure S1g). The trace mode closely follows the contours of the non-ideal cell; however, irregularity of shape and contour of the non-ideal cell may be accentuated. Hand-tracing of the non-ideal cell tends to be dependent on the user’s assessment of where the true boundaries of the adipocyte exist.

## Discussion

Analysis of adipocyte tissue in breast cancer surgical specimens has the potential to contribute to better understanding of host factors important in determining breast cancer outcomes. In addition to other factors such as BMI and macrophage infiltration, useful metabolic and inflammatory information can be gathered from an individual’s adipose histology, which can also be collected with routine breast biopsies during standard surgical pathology methods and are inexpensive to assay. Adipose tissue permits quantitative, morphological and visual analysis, which may reveal further insights into a patient’s overall risk factors. For example, adipocyte size distribution and macrophage infiltration [33–35] are associated with cardiovascular disease and insulin resistance in obesity. Morphological features associated with enlarged adipocytes such as ‘crown-like structures’ [36] are indicative of adipocyte remodelling and concomitant macrophage invasion. These features promote angiogenesis and vascular changes, and an array of immunometabolic responses within adipose tissue, which associate with elevated aromatase expression and may also elevate breast cancer risk [14,37–39]. Thus, visual analysis of adipocyte samples can suggest inflammatory and metabolic complications associated with obesity, which can serve as valuable, systemic health indicators [40] and provide additional clinical parameters that could aid in personalized diagnosis [16,24].

**Figure 7.**
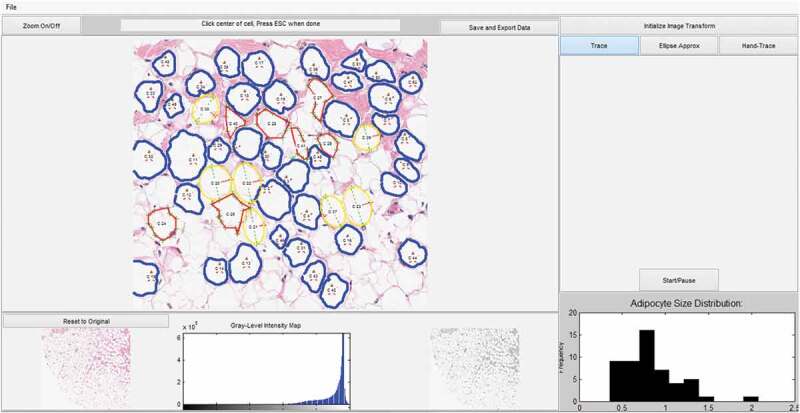
Multiple methods of adipocyte quantification. Challenges of intra-sample variability can be solved for utilizing multiple methods of adipocyte quantification. Due to intra-sample variability, adipocytes quantified using TM are shown outlined in blue, adipocytes quantified using EA are shown outlined in yellow, and adipocytes quantified using HT are shown outlined in red

The primary objective of this work was to develop a new semi-automated method for adipocyte quantitation that demonstrates efficiency and accuracy of adipocyte quantification across multiple sample type, and is better than conventional methods. For the purposes of quantifying adipocytes to determine size distribution as a supplemental assessment of risk, speed and precision of analysis are both highly relevant and critically important. The secondary objective was to develop a full-service software tool for users to analyse a variety of adipocyte samples with the ability to choose different methods as needed. Results of testing show that the AdipoCyze tool was able to quantify adipocyte samples using a variety of quantification methods, including the ability to leverage multiple methods in a single analysis as shown in Figure 7, based on the conditions of the sample being analysed. While intra-sample heterogeneity of quality presents challenges in computationally increasing the efficiency of adipocyte quantification, the selection of methods using AdipoCyze offers a viable solution as demonstrated in Figure S1.

We compared the novel semi-automated trace mode (TM) method with the hand trace (HT) and ellipse approximation (EA) methods. We considered the HT method as the reference method throughout the analysis. As described above, a comparison of the semi-automated trace mode (TM) and emerging fully automated methods was not conducted here, as emerging methods are not considered standard laboratory practice.

Precision of methods was assessed by measuring the variation in the quantification of cells over three repeated runs, as demonstrated in error bars in Figure 3. Variability among trials using the TM was non-existent. Because the semi-automated recursive tracing algorithm does not discriminate on where the user clicks within the cell, as long as the cursor is within the adipocyte cell membrane, the traced region and calculated area will be the same. Thus, the finding of no variability between runs is expected. Conversely, variability run-over-run was consistently the greatest with the EA method, which can reasonably be contributed to the subjectivity involved in assessing the major and semi-minor axes of adipocytes, and the non-exact nature of the approximation. It is reasonable to suggest that there may be a correlation between variability in measurements using the EA method and ovality of the morphological shape of the adipocyte. The HT method that was used as the reference for each cell consistently demonstrated lower, but detectable, variability across samples. This finding is expected, because the HT method introduces human subjectivity and error, yet enables close contouring of the cell perimeter.

In order to compare the accuracy of the three methods of adipocyte quantification, we examined the difference between average measurements per cell number. Using the HT method as a reference (Figure 3), it appears that the TM method consistently underestimates the areas of each cell while the EA mode may under-estimate or over-estimate cell area. The observation of trends of the accuracy of the EA method is likely tied to the previously stated suggestion of correlation with the regularity of adipocyte size, shape, contour or ovality. The consistent underestimation of adipocyte area by the TM method can be explained by appreciating that adipocyte areas generated from the HT method are also an approximation of the true area of the cell and that it is possible that the TM method is more accurate to true cell size than the HT method used here as a reference.

The leftward shift of adipocyte size distribution of the TM method relative to HT and EA is consistent with the finding that TM underestimates adipocyte size relative to HT. However, the general shape and features of the size distribution aligned across the methods. This analysis may benefit from a larger sample size to eliminate possible biases from a limited number of histogram bins. Concordance exists across methods, however results from the tracing algorithm demonstrated that HT and EA methods tend to over-estimate cell size relative to TM as suggested by the linear intercepts, while concordance between HT and TM appears to be strongest.

Analysis rate and overall speed were important metrics for evaluation. The TM method demonstrated the fastest rate of analysis compared to the HT and EA methods. It is noteworthy that the TM rate of analysis poorly fits a linear regression, suggesting a non-linear equation. This can be explained by the additional time required at the beginning of the analysis to transform the image, while subsequent measurements are made very quickly. While it requires about double the amount of time to analyse the first cell using the TM method as HT and EA methods, the TM method completed the analysis in nearly half the time of the EA method and less than one-quarter of the time of the HT method.

Results of comparing the three methods demonstrated that the semi-automated tracing algorithm improved speed, accuracy and precision compared to conventional methods, and preserved adipocyte size distribution. Our work demonstrates that the AdipoCyze tool offers a viable option to quantify adipocyte samples and improves overall efficiency, accuracy and precision, while providing flexibility to work with a wide range of histological sample types as shown in Figure 7. A side-by-side analysis of an ideal cell and a non-ideal cell situated adjacent to each other from an adipocyte histological image sample in Figure S1 revealed that the TM method was able to measure both cells, however as the contours of the cell membrane become irregular, deteriorated or subject to artefacts, the calculated regions begin to differ between methods of analysis. This analysis highlights a limitation of the TM method that researchers should be aware of but also highlights the importance of a tool that offers flexibility to account for the heterogeneity present in adipocyte histological image samples.

AdipoCyze is the first adipocyte-specific image quantification platform to provide the investigator with multiple methods of quantifying adipocytes in a single analysis (Figure 7). Widespread laboratory and clinical use of this program may save time for researchers, while preserving accuracy at acceptable precision, to give insight into patients’ metabolic state. The potential clinical impact of this approach, as well as continued development of the AdipoCyze program, may improve our ability to provide important information on metabolic health, cardiovascular and/or cancer risks, and supplement risk-stratification for more patients. Further characterization of the TM method, such as a direct comparison with emerging fully automated methods, further analysis of adipocyte histological image samples with smaller adipocytes (<2000 µm^2^) and a calculation of a fitment factor to fit TM and HT measurements is warranted. Additional investigation and future development of this method, such as incorporating contemporary techniques of advanced computer vision, machine learning and artificial intelligence approaches, are needed to further enhance and automate adipocyte quantification. Additionally, combining adipocyte size distribution analysis with other clinical and non-clinical parameters, morphological features including crown-like structures, and other factors into a single platform may aid in more comprehensively understanding metabolic status for patients.

The population in which the tool was developed was comprised only African-American women, who had different degrees of obesity and metabolic health. Future validation studies must include other races and test the limits of the tool with a range of subject ages, and in different institutions where the patient population is heterogeneous. It is also possible that medications for Type 2 diabetes, such as metformin or glitazone combinations, which are commonly prescribed for U.S. patients to improve glucose control and adipogenesis, may change adipocyte sizes in ways that the tool can detect, but this prediction must be tested rigorously. Finally, adipocyte size distribution is related to metabolic health in tissues other than breast, such as subcutaneous, omental and visceral adipose depots, in both male and female subjects. A full range of adipose depots must be tested, including for the understudied cases of male breast cancer, a typically aggressive disease, where mechanisms of inflammation, adipocyte size distribution and tumour progression are unknown. Although we have focused here on applications to the microenvironment of women with breast cancer, we point out that AdipoCyze is a generic tool that can be used to quantify adipocyte size in any adipose tissue. Breast adipose tissue biopsies are not performed as the standard of care for metabolic assessment in women without breast cancer or suspected breast cancer. As the relationships between cardiometabolic risk, obesity and cancer outcomes are better understood, broader understanding of the size distribution and morphological characteristics of adipocytes in different adipose depots may prove to have diagnostic and prognostic value.

## Supplementary Material

Supplemental Material
