# Supplementary figures and images for: Novel semi-automated algorithm for high-throughput quantification of adipocyte size in breast adipose tissue, with applications for breast cancer microenvironment
Source: Adipocyte. 2020 Jul 7;9(1):313–25. doi: 10.1080/21623945.2020.1787582 (PMC7469507; doi:10.1080/21623945.2020.1787582)

**Fig. S1: Measurement of Ideal and Non-Ideal Adipocyte Images**

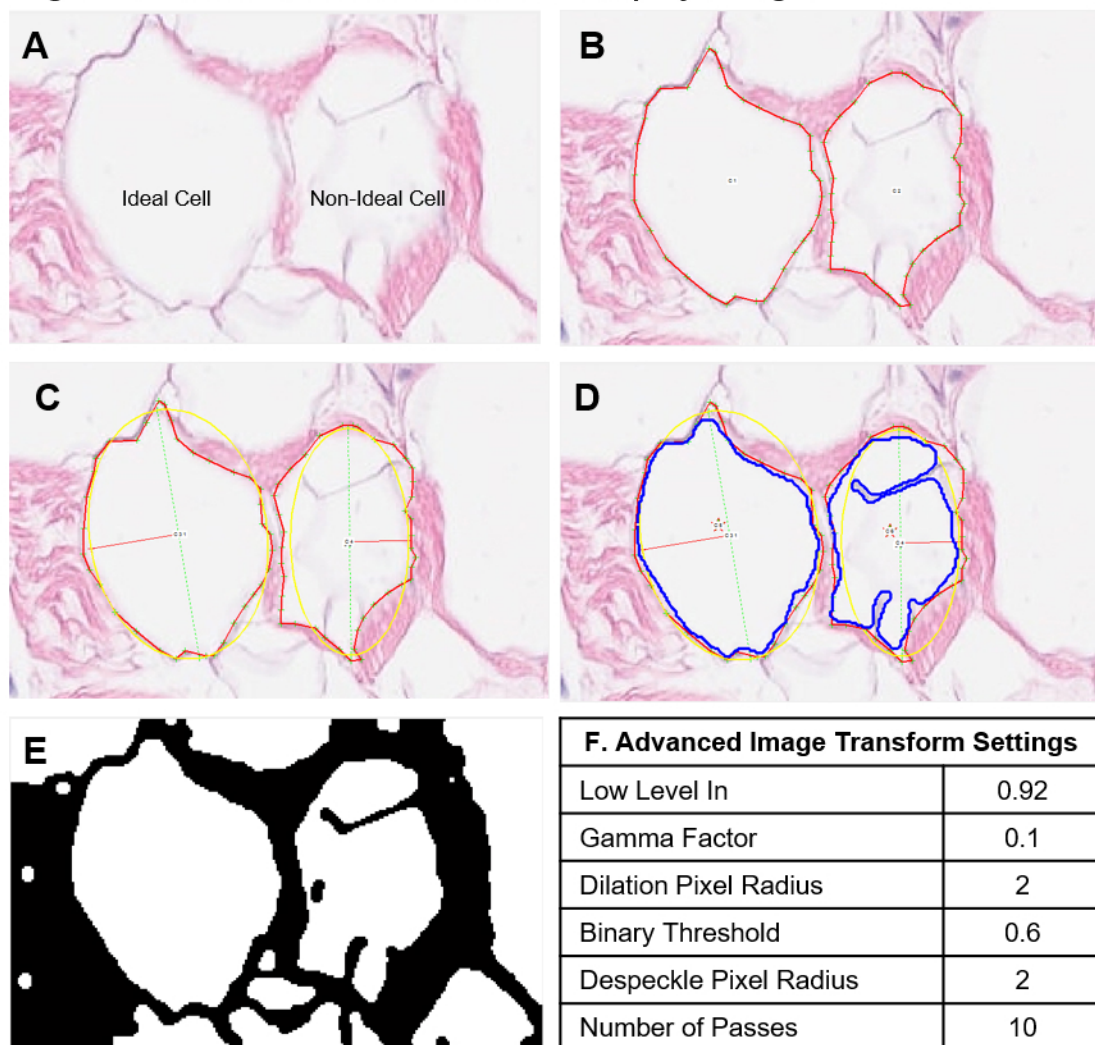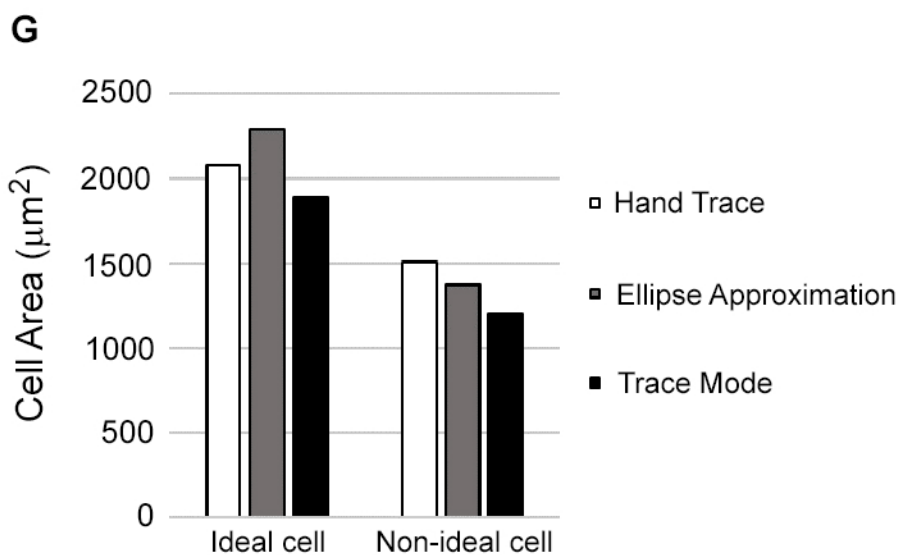

Supplement: Supplemental Material [file KADI_A_1787582_SM2596.pdf]
